# Traumatic experiences and re-victimization of female inmates undergoing treatment for substance abuse

**DOI:** 10.1186/1747-597X-10-5

**Published:** 2015-02-09

**Authors:** Bertha Mejía, Paloma Zea, Martha Romero, Gabriela Saldívar

**Affiliations:** Monte Fénix. Las Flores 439. San Angel Inn. Delegación Alvaro, Delegación Alvaro Obregón, 01060, D. F México; Instituto Nacional de Psiquiatría Ramón de la Fuente Muñiz, Camino a Xochimilco 101. San Lorenzo Huipulco, Tlalpan, 14370, D. F México

**Keywords:** Women, Prison, Substance abuse, Traumatic experiences

## Abstract

**Background:**

In the past decade, several studies have focused on the treatment needs of female inmates with substance abuse problems. An important finding has been that these women are more likely to report histories of sexual, physical, and emotional abuse-at rates varying from 77% to 90%. The trauma resulting from this kind of abuse is a key contributing factor in behavioral problems in adolescence and subsequent delinquency, substance abuse, and criminality in adulthood.

**Methods:**

This was a retrospective clinical study. A convenience sample of 112 women who entered the program’s treatment groups consecutively for one year form part of the study. Information on traumatic events was obtained using some questions from the Initial Trauma Review. It explores whether the participant experienced physical abuse, sexual abuse, disasters, automobile accidents, or witnessed violence under the age of 18. It also examines experiences as an adult, including sexual and physical abuse, attacks by others who are not intimate partners, and abuse by authorities.

**Results:**

Revictimization in sexual abuse was found in 78.1% of participants. Significant differences were identified between women who had experienced a traumatic sexual event from a person five years their senior before the age of 18 and then suffered from sexual violence as an adult, and women who had never undergone either of these events (x^2^ = 11.3, df 112/1, p = <.001). In physical abuse, the figure was 82.17%. Differences were observed between women who were revictimized through physical abuse before and after the age of 18 (x^2^ = 5.91, df 112/1, p = <.01), and those who had not experienced any kind of revictimization. Significant differences were found between women who had suffered a traumatic sexual event as a child and subsequently physical violence from their parents, and women who had not undergone either of these events (x^2^ = 3.48, df 112/1, p = <.05).

**Conclusions:**

Investment in treatment in these areas during the prison sentence and after release may contribute to preventing these women from become repeat offenders. Creating sources of work and halfway houses that continue the program to prevent relapses into substance use can help defend the human rights of this group of women and achieve social justice.

## Background

In the past decade, several studies have focused on the treatment needs of female inmates with substance abuse problems. An important finding has been that these women are more likely to report histories of sexual, physical, and emotional abuse-at rates varying from 77% to 90%. The trauma resulting from this kind of abuse is a key contributing factor in behavioral problems in adolescence and subsequent delinquency, substance abuse, and criminality in adulthood. Moreover, surveys of female inmates have consistently shown a strong link between childhood abuse and mental health problems in adulthood, particularly depression, post-traumatic stress, panic attacks, and eating disorders. The costs of failure to diagnose and treat psychiatric disorders in female criminals are high and may lead to other problems, including unemployment, homelessness, and losing custody of children [1–5].

Research has also shown that female inmates with psychoactive substance dependence are more likely to have chronic health problems, including hepatitis, toxemia, anemia, hypertension, diabetes, and obesity [6]. They also have a greater risk than men of having HIV/AIDS on admission to prison, due to their greater participation in prostitution.

Trauma has been defined as a form of damage to a person that occurs as a result of a severely distressing experience. According to SAHMSA [7], individual trauma results from an event, series of events or set of circumstances experienced by an individual as physically or emotionally harmful or threatening, which has lasting adverse effects on the individual’s functioning and physical, social, emotional or spiritual well-being. According to WHO [8], traumatic events and loss are common in people’s lives.

In a study conducted by the World Health Organization, over 10% of respondents reported witnessing violence (21.8%) or experiencing interpersonal violence (18.8%), accidents (17.7%), exposure to war (16.2%) or a trauma suffered by a loved one (12.5%). An estimated 3.6% of the world population has suffered from posttraumatic stress disorder (PTSD) in the previous year [9]. Violence and trauma, however, have far-reaching effects on the well-being of their victims, particularly in relation to drug use.

The links between physical and sexual abuse, mental illness, and substance abuse are complex, multifaceted, and multidirectional [10]. According to Wilson [11], the experience of women who have been sexually abused in childhood is expressed in several ways: a) behavior, such as dysfunctional relationships, aggression, hostility, substance abuse, and promiscuity; b) emotions, such as fear, anxiety, anger, depression, and hyper vigilance; c) systemic disorders that translate into respiratory, gastrointestinal, neurological, and gynecological problems; and d) pain, including headaches, back pain, and pelvic pain, coupled with the abuse of analgesics. Sexual abuse also leads to the development of consequences of post-traumatic stress, such as insomnia, memory loss, fatigue, and autoimmune disease [12, 13].

An experience of physical, sexual, or emotional abuse may increase the risk of developing mental health problems. In turn, these problems together with substance abuse put women at greater risk of suffering this type of abuse. Lastly, excess consumption and other self-destructive behavior may be the result of traumatic experiences [14]. In other words, there is evidence that the relationship is bidirectional. Sexual abuse generally precedes first alcohol use, although trauma survivors report abuse both before and after. At the same time, childhood sexual abuse is associated with greater substance abuse, which may be linked to an increased risk of revictimization [15].

Whereas victimization is conceived of as receiving violence (physical, sexual or emotional), revictimization is defined as being the victim of interpersonal violence on two or three occasions, carried out by at least two difference perpetrators. More specifically, it refers to undergoing physical or sexual abuse by a family member during childhood, then experiencing it again as an adult, when the perpetrator is the male partner [16].

According to Matthews [17] in a work proposal to reform Latin American prisons, the close relationship between drug use and committing crimes is one of the main problems faced by correctional facilities. Imprisonment can even create or exacerbate drug addiction. In a study on Mexican prisons, Bergman and Azaola [18] report that the prison authorities estimate that 50% of the population regularly consume drugs.

Despite of the previous data, Mexico City's jails (the Centros de Readaptación Social, or CERESOs) have no publicly-funded treatment programs for substance abuse, and there is little available scientific research on the needs of their female inmates [19]. The Monte Fénix Foundation has developed the Recovery from Addiction to Psychoactive Substances Program at the women's CERESO at Santa Martha Acatitla, and covers the full cost of the program. Its objective is to provide a response to this health problem with a program specifically designed for the female population. It grants women access to professional treatment, providing them with information and tools, allowing them to begin a recovery process that leads to a better quality of life.

Among the research questions behind the study were: What are the characteristics of addictive substance use in women? What kind of traumatic experiences have they suffered? Have they experienced revictimization?

The objective of the present study is to describe the traumatic events and revictimization suffered being children and later being adults by the population that entered treatment for abuse of psychoactive substances. Besides, provides recommendations for optimizing their substance abuse and violence treatment.

## Methods

This was a retrospective clinical study. The research project was approved by the Ethics and Research Committee at the Monte Fénix Higher Education Center. In view of the fact that women in prison are a vulnerable group, each woman signed an informed consent form that explained the objectives of the project and of their participation. Moreover, the form clearly stated that there was no risk involved, and that it was a voluntary process that ensured anonymity and confidentiality. To guarantee that there was no coercion, each woman signed her consent form in the presence of two witnesses of their choice, and was informed that if at any moment she decided to drop out of the project, there would be no repercussions on her treatment.

The free treatment model is implemented inside the prison and has the capacity to provide 30 places for each group of women wishing to take part voluntarily. These participants spend 90 days isolated from the rest of the prison population. Thus, every three months a group of 30 women is admitted to these facilities.

A convenience sample of 112 women who entered the program’s treatment groups consecutively for one year form part of the study.

### Treatment

The program uses a facility inside the prison, provided by the CERESO authorities so the participants can be isolated from the rest of the population. The treatment unit was created inside a section of a CERESO and comprises 13 cells. Each of the cells has four beds, a bathroom and a laundry room. The dining area is a space adapted in the corridor, with tables and chairs. It is important to mention that in Mexico there are no mixed gender prison settings.

It has a staff of five therapists and one physician with experience in the treatment of addiction problems, with three additional professionals assigned to provide continuity, who are trained at the Monte Fénix Higher Education Center, at the Clínica de Atención Integral en Desintoxicación y Recuperación (CLAIDER), and on site.

As each woman is admitted, she undergoes a medical clinical assessment in the presence of a nurse. Laboratory tests with 28 elements are taken. The women undergo a pharmacological detoxification process that uses neuroprotective agents. All women with a non-psychotic psychiatric disorder for which they take medicine will continue to take it, in consultation with a psychiatrist.

The program is based on six fundamental concepts:

1. Abstinence: The Path to Recovery
2. Medical Treatment
3. Psychotherapeutic Treatment
4. Education and Information
5. Twelve-step Program of Alcoholics Anonymous
6. Continuous Treatment

Between May 2006 and April 2013, 789 women voluntarily entered the treatment center.

Women were invited to register for a detoxification program located inside the prison. After giving their written informed consent to enter treatment, they signed a written agreement outlining the rules of participation in the program, and lastly an informed consent form, with two witnesses, to allow their information to be used for research purposes.

Treatment takes place outside the general population area, inside the prison and lasted three months. It continues after their return to the prison routine with a relapse prevention program. Participants were subsequently evaluated for follow-up at three and six months, with a urine drug test during the second evaluation. Each treatment group included approximately 30 women. Participants were allowed to leave the group if they wished. Those who did not follow the rules were eliminated. Treatment takes place inside the prison, where psychoactive substances and occasionally alcohol, are available.

### Instrument

To obtain a profile of the participant group on entering treatment, the women were given face-to-face interviews given their literacy skills, using a participant data form. Interviews were conducted by clinical specialists in addiction. The data form included eight sections:

1. Demographic section: age, date of birth, ethnicity, place of birth, length of residence at home.
2. Family and marital history: whether the participant had lived with her biological parents prior to the age of 18, who she was raised by, death of parents, number of biological siblings, sexual orientation, marital status, number of marriages, and number of children.
3. Education and work history: educational attainment and age at which they completed their education, occupation, employment, income, economic dependents.
4. Legal history: arrests, age at first and most recent arrest, months in prison, drug-related crimes, violent crimes, property crimes, public order crimes, other crimes. Criminal activity before and after 18.
5. Psychiatric and medical history: number of times hospitalized for medical and psychiatric problems and age at hospitalization; disorders, age of onset, and medication.
6. Substance abuse
7. A. History of alcohol use
8. B. History of drug use
9. C. Alcohol and drug treatment
10. D. Family history of alcohol and drug problems
11. Traumatic events: Information on traumatic events was obtained using some questions from the Initial Trauma Review, a semi-structured interview that enables the clinician to evaluate the principal forms of exposure to trauma. It explores whether the participant experienced physical abuse, sexual abuse, disasters, automobile accidents, or witnessed violence under the age of 18. It also examines experiences as an adult, including sexual and physical abuse, attacks by others who are not intimate partners, and abuse by authorities [20].
12. Legal situation: length of sentence links between sentence and alcohol or drug use, legal advice, previous imprisonment, age at first imprisonment, family history of legal problems.

The questions used have been validated in Mexican studies, particularly those examining sexual abuse before and after the age of 18 [21].

## Results

A total of 132 women participated in four treatment groups. In the course of the treatment program, 49 women dropped out of the program for a variety of reasons: 12.2% left voluntarily, 16.6% were removed for violation of the rules, 3.03% were released from prison, and 3.7% were transferred to another facility. The study was carried out on the 112 women who provided complete information in the initial interview. Their sociodemographic characteristics are given in Table 1.

**Table 1:** **Demographics**

	N	%
**Civil status**		
Married	16	43.0
Living together	33	29.5
Widowed	5	4.5
Divorced	1	0.9
Separated	20	17.9
Never married/single	37	33.0
**Number of children**		
0	10	9.9
1 -3	68	67.3
4-6	23	22.8
**Sexual preference**		
Heterosexual	60	53.6
Homosexual (gay/lesbian)	12	10.7
Bisexual	39	34.8
Other	1	0.9
**Lived with both biological parents before 18 years**		
Yes	66	58.9
No	46	41.1
**Who raised you before 18 years**		
Both biological parents	31	27.7
Only biological mother	40	35.7
Only biological father	6	5.4
Grandparents	24	21.4
Other relatives	4	3.6
Institution or orphanage	1	0.9
Other	6	5.4

In addition to the characteristics displayed in the tables, 34.8% of the women stated that they suffered from a chronic medical problem. Among the most frequent were those linked to the digestive system (gastroenteritis, hepatitis, cirrhosis, colitis, diabetes), reported by 14 women (17.8%) and gynecological problems (human papilloma virus, myomas and infections) and reported by seven women (8.9%). Only two women (2.6%) reported that they took psychiatric medicine.

The criminal offenses for which they were imprisoned were mainly various kinds of theft (65.8%), in addition to murder (7.1%); crimes against public health (5.6%); kidnapping (7.4%); extortion, child abduction, murder of a relative, and other crimes (11.4%) together with a smaller number of crimes involving forgery and organized crime and kidnapping (1.8%). Table 2 shows substance use and average age of initial use. After alcohol and tobacco consumption, the most frequently used drugs were marijuana, cocaine, and inhalants.

**Figure 1:**
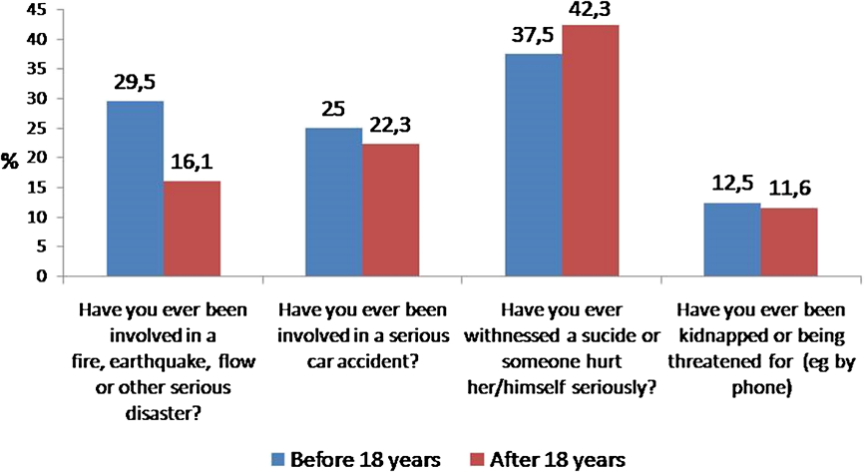
**Disasters, accidents, witnessed suicide, kidnapping.**

Figure 1 shows the percentage of women who experienced trauma such as disasters, serious automobile accidents, witnessing suicides, or kidnapping threats. It should be noted that the last two were common experiences being adults. Of particular interest are the high percentage of those who experienced trauma before the age of 18 and those who witnessed a suicide after that age.

**Figure 2:**
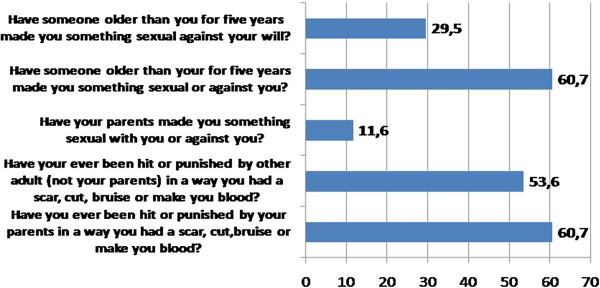
**Physical and sexual abuse before 18 years.**

Figure 2 shows physical and sexual abuse experienced prior to the age of 18.

**Figure 3:**
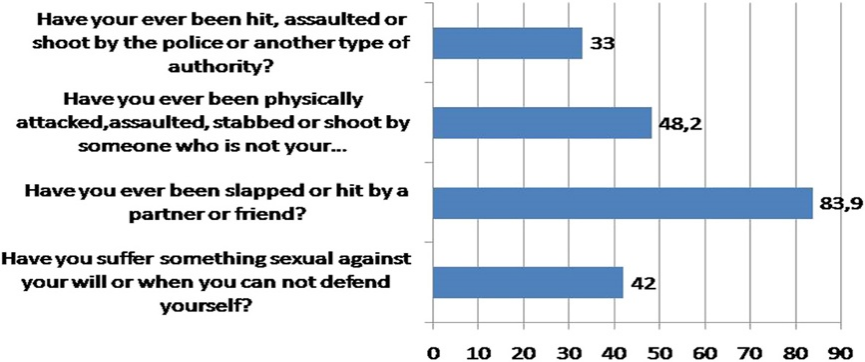
**Adult physical and sexual abuse N = 112.**

Figure 3 shows adult physical and sexual abuse.

**Figure 4:**
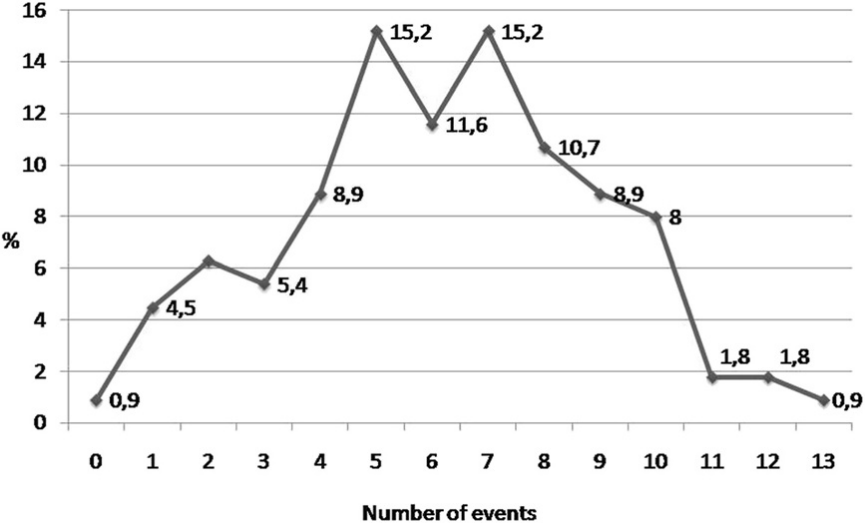
**Traumatic events distributions.** N = 112, X̄ = 6.2.

Due to the fact that a single woman may have suffered several traumatic events, it was decided that both the distribution and an average of these would be calculated. Figure 4 shows the distribution of traumatic events in the study group, with a range of 1–13 events and a mean of 6.

**Table 2:** **Lifetime drug use**

	Yes		No		Age of onset (Media and deviation)
	N	%	n	%	
Alcohol	109	(97.3)	3	(2.7)	14.90 (5.61)
Tobacco	110	(98.2)	2	(1.8)	14.99 (5.90)
Marihuana	101	(89.3)	11	(10.7)	20.16 (8.32)
Cocaine	95	(83.9)	17	(16.1)	24.13 (32.07)
Heroin	9	(7.2)	103	(92.8)	17.89 (9.04)
Hallucinogens	20	(18.9)	92	(81.1)	17.75 (5.57)
Inhalants	63	(57.1)	49	(42.9)	17.08 (6.60)
Amphetamines	22	(19.3)	90	(80.7)	20.77 (7.67)
Opiates	2	(0.9)	110	(99.1)	22.50 (3.53)
Tranquilizers	54	(49.5)	58	(50.5)	21.00 (6.06)
Sedatives	5	(4.8)	107	(95.2)	21.40 (6.58)
Stimulants	4	(3.7)	108	(96.3)	21.25 (9.50)
Other drugs	11	(15.9)	101	(84.1)	20.0 (9.06)

To undertake an analysis of the link between demographic variables and the number of lifetime traumas, women were divided into two groups: one group with 0–6 traumatic events and another with 7 or more. The variables analyzed were marital status, sexual orientation, death of parents under the age of 18, who the participant was raised by, whether her parents were divorced, and whether she had lived with both parents at some time under the age of 18. Only the last of these variables proved significant. Women who had lived with their biological parents constituted a greater proportion of the group with 0–6 traumatic events (x^2^ = 5.7, df = 1, p < .01); in other words, those who had lived with their families experienced fewer such events.

To analyze revictimization in sexual and physical abuse, without regard to the identity of the perpetrator, variables were crossed while being child and like an adult. Revictimization in sexual abuse was found in 78.1% of participants (Table 3). Significant differences were identified between women who had experienced a traumatic sexual event from a person five years their senior before the age of 18 and then suffered from sexual violence as an adult, and women who had never undergone either of these events (x^2^ = 11.3, df 112/1, p = <.001).

**Table 3:** **Sexual abuse revictimization**

Sexual abuse	Yes adult	No adult
**YES childhood**	37 (78.7%)**	31 (47.7%)
**NO childhood**	10 (21.3%)	34 (52.3%)
	47 (100%)	65 (100%)

**(x^2^ = 11.2, gl 112/1, p = <.001).

In physical abuse, the figure was 82.17%. Considerable differences were observed between women who were revictimized through physical abuse before and after the age of 18 (x^2^ = 5.91, df 112/1, p = <.01), and those who had not experienced any kind of revictimization (Table 4).

**Table 4:** **Physical abuse revictimization**

Physical abuse	Yes adult	No adult
**Yes childhood**	83 (82.17%)*	5 (45%)
**No childhood**	18 (17.83%)	6 (54%)
	101 (100%)	11 (100%)

*x^2^ = 5.91, df 112/1, p = <.001.

Given that a woman may experience both physical and sexual abuse during childhood (before the age of 18), variables were crossed: Before you were 18, did either of your parents hit you or punish you in a way that left a bruise, cut or scar, or made you bleed? With the variable, Has a person at least five years older than you done anything sexual with or towards you? Significant differences were found between women who had suffered a traumatic sexual event as a child and subsequently physical violence from their parents, and women who had not undergone either of these events (x^2^ = 3.48, df 112/1, p = <.05) (Table 5).

**Table 5:** **Traumatic physical and sexual events in childhood**

Before 18. Have you ever been hit or punished by your parents in a way you had a scar, cut, bruise or make you blood?	Before 18. Have someone older than you for five years or more made you something sexual against your will?	
	Yes	No
**Yes**	46 (67.6%)*	22 (32.4%)
**No**	22(50%)	22(50%)

*(x^2^ = 3.48, df 112/1, p = .05).

A logistic regression analysis was not possible, as the study sample is extremely homogenous. Neither were notable differences found between revictimization and the use of specific psychoactive substances.

An analysis of the cases in which follow-up revealed a positive drug test compared with negative tests showed no significant differences in the violent events reported.

## Discussion

There is no doubt that the women who participated in this study are a population that has experienced multiple traumas. Messman-Moore, Walsh, and DiLillo [22] define revictimization as an elaboration of the psychological impact of previous victimization. It is associated with increased illness, including high anxiety levels, current and lifelong depression, heavy drinking, and past month drug use. According to these authors, the majority of revictimization models focus on how post-traumatic sequelae associated with sexual abuse influence psychological, cognitive, and interpersonal functioning, thereby increasing the risk of revictimization. In this study, 78.7% of the participants reported sexual abuse revictimization. This figure is higher to the figure of 38% reported by Walsh, DiLillo, and Scalora [23] in a study of female inmates. Childhood abuse also has a negative impact on the development of the capacity to regulate the emotions, while difficulty using emotional regulation increases a person's vulnerability to psychological disorders. In this study, 82.17% of participants reported physical abuse revictimization. Victimized women are often afraid of being newly victimized during the process of seeking help [24].

One of the limitations of this study is the lack of a control group of female inmates who were not substance abusers. This type of control would be required to determine whether the level of violence experienced by substance abusers was greater than that of the rest of the population. However, having a control group with a doubly vulnerable population in a marginalized situation of social exclusion would pose an ethical dilemma, since undertaking research with a group of women who do not receive any benefit in exchange for their participation could be regarded as an abuse of power.

In the case of women in prison, penitentiary policies and administration should encourage the implementation of social readaptation and reinsertion programs, by reducing marginalization and providing treatment. The majorities of female prisoners belong to less privileged social classes and have limited cultural knowledge, in addition to problems of substance abuse and, as shown in the study, various experiences of traumatic violence. Investment in treatment in these areas during the prison sentence and after release may contribute to preventing these women from become repeat offenders. Creating sources of work and halfway houses that continue the program to prevent relapses into substance use, which foster self-care habits and provide reproductive health programs and specialized treatment for trauma and violence, can help defend the human rights of this group of women and achieve social justice.

## Authors’ information

BM Addiction specialist. She is the general director of Monte Fénix Clinic and has been in charge of addiction treatment supervision for the past 20 years. She designed the prison program for women inmates to contribute to crime prevention and inmates’ drug rehabilitation.

PZ Addiction specialist. She was the director of the Higher Education Center of Monte Fénix when the research was conducted. She was in charge of the addiction specialization studies for training health professionals.

MR holds an MD in clinical psychology and a PhD in anthropology. She is a medical science researcher at the National Institute of Psychiatry and her research interests include women’s addiction problems. She is a scientific advisor to Monte Fénix.

GS She holds a PhD in psychology. She is a medical science researcher at the National Institute of Psychiatry and specializes in sexual violence problems.
